# Effectiveness of the low FODMAP diet in patients with irritable bowel syndrome and small intestine bacterial overgrowth syndrome

**DOI:** 10.3389/fnut.2026.1725524

**Published:** 2026-01-28

**Authors:** Dagmara Bogdanowska-Charkiewicz, Piotr Górski, Grażyna Jurkowska, Roksana Środa, Agnieszka Świdnicka-Siergiejko, Andrzej Dąbrowski, Jarosław Daniluk

**Affiliations:** 1 Department of Gastroenterology and Internal Medicine, Medical University of Bialystok, Bialystok, Poland; 2 Klinika MajDiet, Dąbrowa Górnicza, Poland

**Keywords:** IMO, intestinal methanogen overgrowth, irritable bowel syndrome, low FODMAP diet, SIBO, small intestine bacterial overgrowth

## Abstract

**Background:**

The low FODMAP diet (fermentable oligosaccharides, disaccharides, monosaccharides and polyols, LFD) t is used in patients diagnosed with Irritable Bowel Syndrome (IBS) as part of non-pharmacological treatment. This approach is also used in patients with Small Intestine Bacterial Overgrowth (SIBO) or Intestinal Methanogen Overgrowth (IMO), but research in this area is insufficient. To fill this gap, we decided to investigate the effect of a low FODMAP diet on various parameters in patients with IBS or SIBO or IMO. The primary end point of the study was to assess the effect of a low FODMAP diet on the severity of gastrointestinal symptoms in patients diagnosed with IBS or SIBO or IMO. Secondary end points included evaluation of patients’ tolerability of the low FODMAP diet; assessment of potential difficulties in completing a low FODMAP diet and evaluation of the role of the dietitian in the implementation of a complete low FODMAP diet.

**Methods:**

The study was conducted using an original unvalidated questionnaire completed online by adult patients diagnosed with IBS according to Rome IV Criteria and/or SIBO/IMO patients diagnosed with hydrogen-methane breath test, treated with a low FODMAP diet.

**Results:**

A total of 98 out of 118 patients who were invited to participate took part in an online survey. The participants had previously followed a low FODMAP diet. 90.7% of IBS patients reported reduction of symptoms after LFD. The greatest reduction in symptom severity before and after the LFD was found for bloating (MD -5.03, *p* < 0.001). Less than half of the patients reported diet as easy to follow and only 43,9% of patients completed LFD. 62.2% of patients were supported by a dietetitian. Patients who completed both phases of the diet were about 3.5 times more likely to improve symptoms. Those of the patients who were taking antibiotics before the diet were approximately seven times more likely to respond well to the low FODMAP diet (OR = 7.10, *p* = 0.028). The beneficial effect of a LFD on symptom reduction was independent of the initial diagnosis. Dividing the diet into elimination and reintroduction phases was not significantly associated with better results (*p* = 0.305). However, completing the entire diet program (i.e., going through both phases: elimination and reintroduction) was significantly associated with greater improvement (OR = 3.43, *p* = 0.024). The use of probiotics during the diet did not have a significant effect on its outcome (*p* = 0.529). No difference was observed between those who took them and those who did not use probiotics. Other factors (age, gender, dietary consultation, diet tolerance) – none of these factors had a significant impact on improvement after the diet – the results did not differ depending on age, gender or whether the patient had a dietary consultation.

**Conclusion:**

Low FODMAP diet was well tolerated and reduced reported gastrointestinal symptoms, with the greatest effect on reducing bloating. The reduction in symptoms occurred independently of the initial diagnosis. Patients who completed both phases of the diet were taking antibiotics before the diet more likely to respond well to the low FODMAP diet. The diet proved difficult to follow, and more than half of the patients sought the support of a dietitian, which confirms the importance of such support.

## Introduction

**Figure 1:**
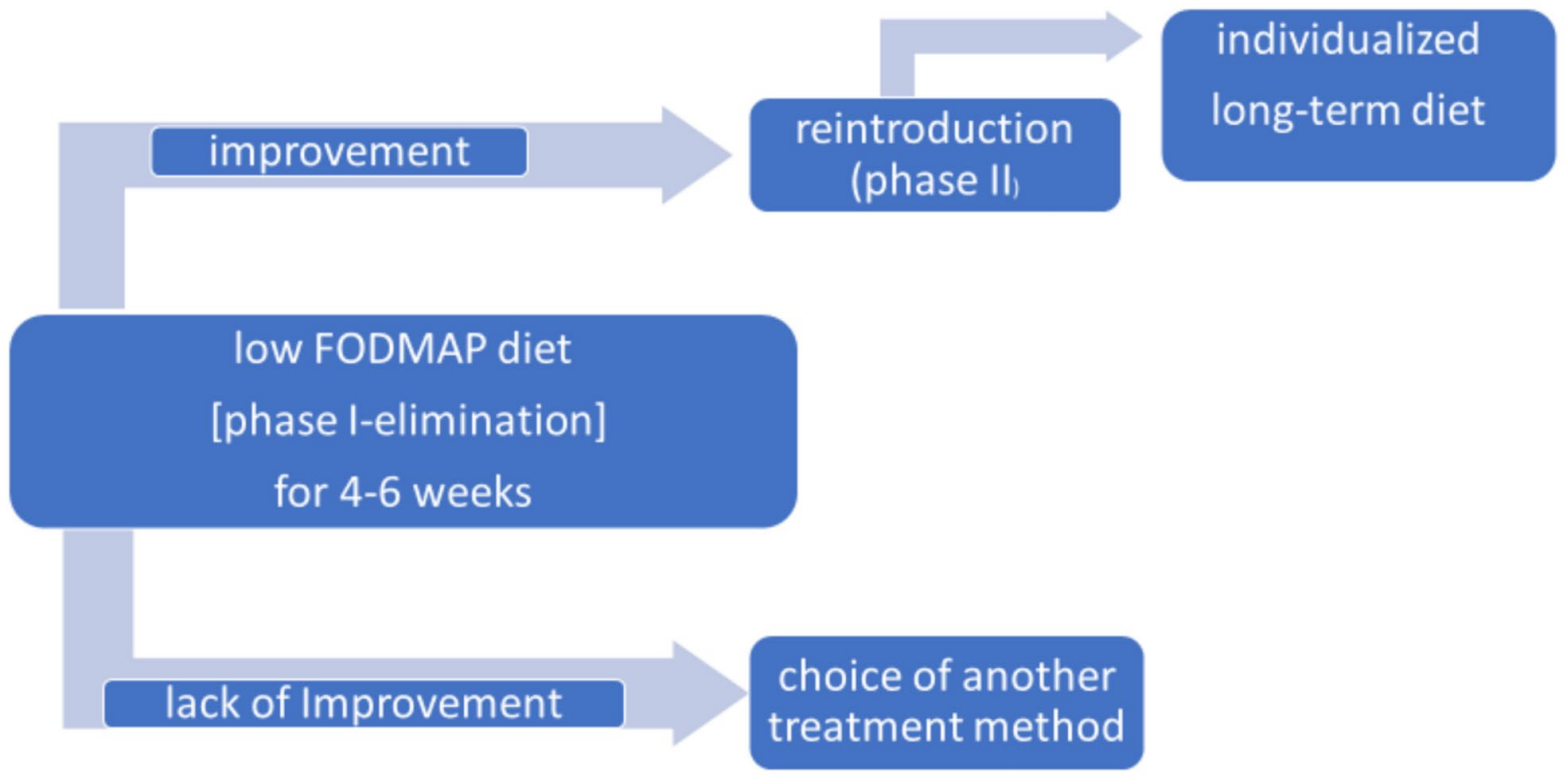
Phases of the low FODMAP diet (57).

FODMAPs stands for Fermentable Oligosaccharides, Disaccharides, Monosaccharides, and Polyols. These substances are commonly found in many food products. Their consumption may exacerbate gastrointestinal symptoms such as bloating, abdominal pain, and fluctuating bowel movements. According to the American Gastroenterological Association (AGA), American College of Gastroenterology (ACG) and British Society of Gastroenterology (BSG), in patients diagnosed with (IBS) the first-line intervention consist of comprehensive patient education with lifestyle changes (exercise, stress-reduction, and diet modification). One of the recommended diet intervention is a low FODMAP diet, i.e., with a restriction of the above foods (Table 1) (1). This diet should be followed for 4–6 weeks (phase I-elimination) and then its effectiveness is assessed (Figure 1). If, during this time, a reduction in abdominal pain and bloating and/or an improvement in bowel movements is obtained, phase II of the diet (reintroduction) should be started, during which the previously excluded products are introduced one by one. Prolongation of the first phase of the low FODMAP diet (LFD) is inadvisable if it is not effective, due to adverse changes to the intestinal microbiota (reduced diversity of bacterial strains, e.g., reduced population of the *Bifidobacterium* strain), the occurrence of deficiencies of vitamins A, D, B12, iron, calcium and worsening of possible malnutrition (2, 3). Dietitian support can be an important part of dietary intervention. Previous studies have shown the effectiveness of the LFD in reducing symptoms in some IBS patients. The lack of full efficacy may be due to inappropriate dietary management or interruption of the diet (4, 5).

**Table 1:** FODMAP contents of food (49).

	Expand the letter from the acronym FODMAP	Substances	Food products
F	Fermentable		
O	Oligosaccharides	Fructans, galacto-oligosaccharides	Wheat, barley, rye, onions, leek, white part of spring onions, garlic, shallots, artichokes, beets, fennel, peas, chicory, pistachios, cashews, legumes, lentils, chickpeas
D	Disaccharides	Lactose	Milk, pudding, ice cream, yogurt
M	Monosaccharides	Fructose	Apples, pears, mangoes, cherries, watermelon, asparagus, sugar snap peas, honey, high fructose corn syrup
A	And		
P	Polyols	Sorbitol, mannitol, maltitol, xylitol	Apples, pears, apricots, cherries, nectarines, peaches, plums, watermelon, mushrooms, cauliflower, artificially sweetened chewing gum and confectionery products

Despite the widespread use of the low FODMAP diet, there are still few data on its effectiveness in IBS and SIBO/IMO (6–9).

Therefore, the aim of our study was to evaluate the effectiveness of the LFD in reducing the symptoms of IBS and/or SIBO or IMO, diet tolerance, and the ability of patients to follow the diet independently or in cooperation with a dietitian.

## Materials and methods

Adult patients of the Gastroenterology Outpatient Clinic of the University Clinical Hospital in Bialystok were invited to participate in the study. All participants were previously diagnosed with IBS according to Rome IV Criteria and/or SIBO or IMO diagnosed with hydrogen-methane breath test. All of them experienced a low FODMAP diet with phase I (elimination) lasting 4–6 weeks and phase II (reintroduction) with a duration depending on the tolerance of the foods introduced.

Patients who had previously agreed to receive information by email during their ambulatory visit to the doctor were invited by email to complete an online survey.

Of the 118 patients who were invited to participate in the study, 98 gave their informed consent to participate in an online survey and completed survey. They did not receive any financial compensation for their participation. The study was conducted from September 2024 to January 2025 and was approved by the Bioethics Committee of the Medical University of Bialystok (No APK.002.601.2024).

The patient could select more than one diagnosis if it was confirmed by a physician in accordance with the inclusion criteria for the study. Patients reported the severity of the symptoms rated on a scale from 0 to 10 points. They reported reduction in specific symptoms after the LFD on the same scale. Total Symptom Burden Score (TSBS) was calculated as the sum of individual symptom severity scores and further used as general indicator of diet effectiveness, along with severity of individual symptoms. Based on the TBSB score participants were classified as responders (reduction of ≥20 points) or non-responders (reduction of <20 points).

### Inclusion criteria and exclusion criteria

#### Inclusion criteria

- adult patients diagnosed with IBS according to Rome IV Criteria
- adult patients diagnosed with SIBO/IMO with hydrogen-methane breath test with 10 grams of lactulose (QuinTron BreathTracker SC)

#### Exclusion criteria

- no consent to complete the online questionnaire

### Statistical analysis

Statistical analysis was performed in R software, version 4.4.2. Continuous variables were expressed as means (±standard deviation) or medians (interquartile range), and categorical variables as counts and percentages. Shapiro–Wilk, skewness and kurtosis were used to assess distribution normality. Levene’s test was used to assess homogeneity of variances.

Pre–post differences were analyzed using the paired t-test or Wilcoxon signed-rank test, depending on normality od difference distribution. Comparisons between responders and non-responders were performed with Student’s t-test or Mann–Whitney U test for numeric variables, and Pearson’s chi-square test or Fisher’s exact test for categorical data, as appropriate. Two step logistic regression was performed to identify predictors of response. Each model was adjusted with TSBS before diet as a covariate. Variables selection to multivariate model was based on the *p* < 0.25^1^ condition (univariate models). Model fit was evaluated with Nagelkerke’s R2 and Hosmer and Lemeshow test, and multicollinearity was assessed using variance inflation factors (VIFs).

## Results

### Study group characteristics

A total of 98 out of 118 patients who were invited to participate took part in an online survey. Baseline study group characteristics is presented in Table 2. 95,9% of participants were women, median age of participants was 33.5 years. Diagnosed diseases for which the LFD was recommended for treatment were SIBO (diagnosed using hydrogen-methane breath test with 10 grams of lactulose) in 82.7%; IMO (diagnosed using hydrogen-methane breath test) in 51%; IBS (diagnosed according to Rome IV Criteria) in 37.8%.

**Table 2:** Baseline study group characteristics and diet process (*n* = 98).

Variable	Statistic
Age, years, Me (IQR)	33.50 (29.00; 39.00)
Sex, female, *n* (%)	94 (95.9)
Disease type, *n* (%)	
SIBO	81 (82.7)
IMO	50 (51.0)
IBS	37 (37.8)
Other	7 (7.1)
Symptoms, *n* (%)	
Abdominal pain	74 (75.5)
Bloating	96 (98.0)
Fluctuating bowel movements	79 (80.6)
Diarrhea	51 (52.0)
Constipation	64 (65.3)
Sudden tenesmus	32 (32.7)
Painful tenesmus	18 (18.4)
Other	7 (7.1)
Symptoms severity	
Abdominal pain [0–10], M ± SD	5.94 ± 3.36
Bloating [0–10], Me (IQR)	9.00 (8.00; 10.00)
Fluctuating bowel movements [0–10], Me (IQR)	6.00 (3.00; 8.00)
Diarrhea [0–10], Me (IQR)	3.00 (0.00; 7.00)
Constipation [0–10], Me (IQR)	6.00 (0.25; 8.00)
Sudden tenesmus [0–10], Me (IQR)	0.50 (0.00; 6.00)
Painful tenesmus [0–10], Me (IQR)	0.00 (0.00; 3.00)
Total Symptom Burden Score (TSBS) [0–70], M ± SD	33.36 ± 11.86
Assessment of general well-being [1–10], M ± SD	3.74 ± 2.74
Antibiotics before diet, *n* (%)	89 (90.8)
Antibiotics before the diet – type, *n* (%) *	
Rifaksimin	37 (41.6)
Rifaksimin and Metronidazole	13 (14.6)
Rifaksimin and Neomycin	39 (43.8)
Diet split elimination and reintroduction phases, *n* (%) **	75 (78.1)
Dietary consultation, *n* (%)	61 (62.2)
Full diet finalized (elimination and reintroduction), *n* (%)	43 (43.9)
Good tolerance of diet, *n* (%)	93 (94.9)
Probiotics during the diet, *n* (%)	61 (62.2)

M, Mean; Me, Median; SD, Standard deviation; IQR, Interquartile range. *Proportions calculated for all patients with antibiotics. **Data available for *n* = 96 patients.

Among respondents, the most common symptoms were bloating (98%), fluctuating bowel movements (80.6%), abdominal pain (75.5%). Interestingly, more patients were suffering from constipation (65.3%) than from diarrhea (52%). In most cases (90.8%) low FODMAP diet was preceded by antibiotic therapy: rifaximin (41.6%); rifaximin and neomycin (43.8%); rifaximin and metronidazole (14.6%).

### Effectiveness of therapy – severity of symptoms before and after the diet

**Figure 2:**
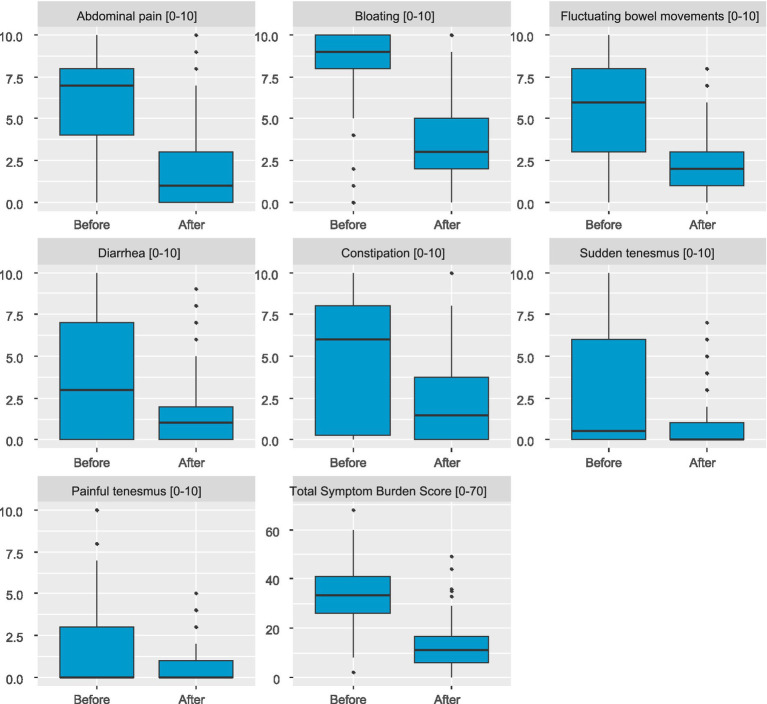
Change in severity of individual symptoms and change in total symptom burden score (TSBS) from before to after the diet.

After following a low FODMAP diet, a reduction in the severity of all assessed symptoms was observed (Table 3): abdominal pain decreased by 3.90, MD = -3.90 CI95 [−4.46;-3.34], *p* < 0.001, bloating by 5.03, MD = -5.03 CI95 [−5.61;-4.45], *p* < 0.001, fluctuating bowel movements by 3.51, MD = -3.51 CI95 [−4.14;-2.88], *p* < 0.001, diarrhea by 2.20, MD = -2.20 CI95 [−2.78;-1.63], *p* < 0.001, constipation by 3.00, MD = -3.00 CI95 [−3.62;-2.38], *p* < 0.001, sudden tenesmus decreased by 1.73, MD = -1.73 CI95 [−2.26;-1.21], *p* < 0.001. Significant decrease was observed also in severity of painful tenesmus, MD = 0.00 CI95 [−1.00;0.00], *p* < 0.001. Total Symptom Burden Score (TSBS) noted significant reduction from 33.36 ± 11.86 before diet to 12.66 ± 9.61 after diet, MD = -20.69 CI95 [−22.67;-18.72], *p* < 0.001 (Table 4; Figure 2).

**Table 3:** Patients’ assessment of diet (*n* = 98).

Variable	Statistic
General improvement observed, *n* (%) *	88 (90.7)
Symptoms improvement observed, *n* (%)	
Abdominal pain	66 (67.3)
Bloating	78 (79.6)
Fluctuating bowel movements	62 (63.3)
Diarrhea	44 (44.9)
Constipation	39 (39.8)
Sudden tenesmus	18 (18.4)
Painful tenesmus	16 (16.3)
Other	4 (4.1)
Diet was easy to use, *n* (%)	43 (43.9)
Would repeat the diet in case of disease recurrence, *n* (%)	89 (90.8)

*Data available for *n* = 97 patients.

**Table 4:** Assessment of symptoms severity before and after the diet (*n* = 98).

Variable	Before diet	After diet	MD (95% CI)	*p*
Symptoms severity				
Abdominal pain [0–10], M ± SD	5.94 ± 3.36	2.04 ± 2.17	−3.90 (−4.46; −3.34)	<0.001
Bloating [0–10], M ± SD	8.31 ± 2.29	3.28 ± 2.38	−5.03 (−5.61; −4.45)	<0.001
Fluctuating bowel movements [0–10], M ± SD	5.55 ± 3.31	2.04 ± 1.87	−3.51 (−4.14; −2.88)	<0.001
Diarrhea [0–10], M ± SD	3.72 ± 3.55	1.52 ± 2.03	−2.20 (−2.78; −1.63)	<0.001
Constipation [0–10], M ± SD	5.17 ± 3.74	2.17 ± 2.35	−3.00 (−3.62; −2.38)	<0.001
Sudden tenesmus [0–10], M ± SD	2.73 ± 3.32	1.00 ± 1.59	−1.73 (−2.26;−1.21)	<0.001
Painful tenesmus [0–10], Me (IQR)	0.00 (0.00;3.00)	0.00 (0.00;1.00)	0.00 (−1.00; 0.00)	<0.001
Total Symptom Burden Score (TSBS) [0–70], M ± SD	33.36 ± 11.86	12.66 ± 9.61	−20.69 (−22.67; −18.72)	<0.001

M, Mean; Me, Median; SD, Standard deviation; IQR, Interquartile range. Data presented as M ± SD or Me (IQR), depending on distribution normality. MD, Mean or median difference (after diet vs before diet); CI, Confidence interval. Comparisons performed with paired t test (unless indicated otherwise) or Wilcoxon test (painful tenesmus).

### Factors of successful diet outcome

**Figure 3:**
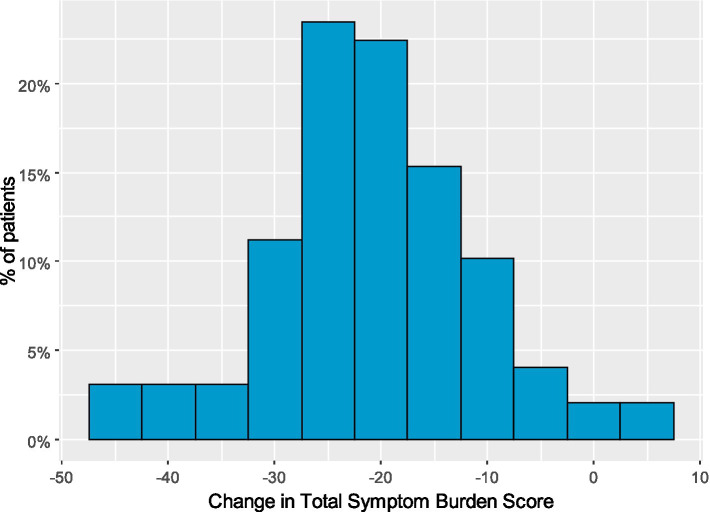
Histogram presenting the distribution of total symptom burder score (TSBS) change from before to after diet.

To investigate the factors increasing successful diet outcome, patients were split into subgroups with better response, called *responders*, while the rest of the group was named *non-responders*. Responders were defined as patients with reduction of TSBS of at least 20 pts. (based on the distribution of the TSBS change, Figure 3). Responders group consisted of *n* = 58 patients with higher response to the diet and non-responders group consisted of *n* = 40 patients with lower response to the diet.

Baseline characteristics were compared between responders and non-responders. Painful tenesmus before diet was 6x more common among responders (27.6% vs. 5.0%, n = 2, RR = 5.52 CI95 [1.34;22.68], *p* = 0.010). Severity of the following symptoms was higher among responders before introduction of the diet: abdominal pain (MD = 2.00 CI95 [0.00;3.00], *p* = 0.012), bloating (MD = 1.00 CI95 [0.00;2.00], *p* < 0.012), fluctuating bowel movements (MD = 2.00 CI95 [1.00;4.00], *p* = 0.002) and painful tenesmus (MD = 1.00 CI95 [0.00;2.00], *p* = 0.008). Total Symptom Burden Score (TSBS) before diet was significantly higher among responders, MD = 10.57 CI95 [6.20;14.94], *p* < 0.001 (Table 5).

**Table 5:** Comparison of patients with higher (responders) and lower (non-responders) response to diet.

Group	Responders (n = 58)	Non-responders (n = 40)	MD/RR (95% CI)	p
Age, years, Me (IQR)	32.50 (28.25; 37.75)	34.50 (30.00; 39.25)	−2.00 (−4.00; 2.00)	0.385
Sex, female, *n* (%)	56 (96.6)	38 (95.0)	1.02 (0.93; 1.11)	>0.999
Disease type, *n* (%)				
SIBO	48 (82.8)	33 (82.5)	1.00 (0.83; 1.21)	>0.999
IMO	28 (48.3)	22 (55.0)	0.88 (0.60; 1.29)	0.654
IBS	26 (44.8)	11 (27.5)	1.63 (0.91; 2.91)	0.127
Other	3 (5.2)	4 (10.0)	0.52 (0.12; 2.19)	0.439
Symptoms, n (%)				
Abdominal pain	48 (82.8)	26 (65.0)	1.27 (0.99; 1.64)	0.077
Bloating	57 (98.3)	39 (97.5)	1.01 (0.95; 1.07)	>0.999
Fluctuating bowel movements	49 (84.5)	30 (75.0)	1.13 (0.91; 1.39)	0.364
Diarrhea	32 (55.2)	19 (47.5)	1.16 (0.78; 1.73)	0.588
Constipation	39 (67.2)	25 (62.5)	1.08 (0.80; 1.45)	0.788
Sudden tenesmus	21 (36.2)	11 (27.5)	1.32 (0.72; 2.42)	0.494
Painful tenesmus	16 (27.6)	2 (5.0)	5.52 (1.34; 22.68)	**0.010**
Other	6 (10.3)	1 (2.5)	4.14 (0.52; 33.07)	0.235
Symptoms severity				
Abdominal pain [0–10], Me (IQR)	7.00 (6.00; 9.00)	5.00 (0.00; 8.00)	2.00 (0.00; 3.00)	**0.012**
Bloating [0–10], Me (IQR)	9.00 (9.00; 10.00)	8.00 (7.00; 10.00)	1.00 (0.00; 2.00)	**<0.001**
Fluctuating bowel movements [0–10], Me (IQR)	7.00 (4.25; 9.00)	5.00 (1.00; 7.00)	2.00 (1.00; 4.00)	**0.002**
Diarrhea [0–10], Me (IQR)	4.00 (0.00; 8.00)	2.00 (0.00; 5.25)	2.00 (0.00; 3.00)	0.127
Constipation [0–10], Me (IQR)	6.00 (1.25; 8.00)	5.00 (0.00; 8.00)	1.00 (0.00; 2.00)	0.296
Sudden tenesmus [0–10], Me (IQR)	1.50 (0.00; 6.00)	0.00 (0.00; 4.00)	1.50 (0.00; 2.00)	0.112
Painful tenesmus [0–10], Me (IQR)	1.00 (0.00; 5.00)	0.00 (0.00; 1.00)	1.00 (0.00; 2.00)	**0.008**
Total Symptom Burden Score (TSBS) [0–70], M ± SD	37.67 ± 10.07	27.10 ± 11.58	10.57 (6.20; 14.94)	**<0.001**
Assessment of general well-being [1–10], M ± SD	3.62 ± 2.75	3.92 ± 2.76	−0.30 (−1.43; 0.82)	0.592
Antibiotics before diet, *n* (%)	54 (93.1)	35 (87.5)	1.06 (0.93; 1.22)	0.480
Antibiotics before the diet – type, *n* (%) *				
Rifaksimin	24 (44.4)	13 (37.1)	–	0.751
Rifaksimin and Metronidazole	8 (14.8)	5 (14.3)		
Rifaksimin and Neomycin	22 (40.7)	17 (48.6)		
Diet split elimination and reintroduction phases, *n* (%) **	47 (82.5)	28 (71.8)	1.15 (0.91; 1.45)	0.322
Dietary consultation, *n* (%)	38 (65.5)	23 (57.5)	1.14 (0.82; 1.58)	0.553
Full diet finalized (elimination and reintroduction), *n* (%)	28 (48.3)	15 (37.5)	1.29 (0.80; 2.08)	0.396
Good tolerance of diet, *n* (%)	56 (96.6)	37 (92.5)	1.04 (0.94; 1.15)	0.396
Probiotics during the diet, *n* (%)	35 (60.3)	26 (65.0)	0.93 (0.68; 1.26)	0.799

M, Mean; Me, Median; SD, Standard deviation; IQR, Interquartile range. Data presented as M ± SD or Me (IQR), depending on distribution normality. MD, Mean or median difference (responders vs non- responders); RR, Relative risk (responders vs non- responders); CI, Confidence interval. Comparisons of numeric variables performed with t-Student test (TSBS, assessment of general well-being) or Mann–Whitney U test (unless indicated otherwise). Comparisons of categorical variables performed with Person’s chi-square test or Fisher’s exact test, as appropriate. Responders defined as patients with TSBS reduction of at least 20 points. *Proportions calculated for all patients with antibiotics. ** Data available for *n* = 96 patients.

Logistic regression analysis for responders (vs non-responders) was performed to identify factors influencing odds of higher response to diet. Level of TSBS before diet was included in all models as a covariate to adjust for differences in general symptoms severity at baseline. Univariate analysis did not indicate significant outcomes. Multivariate model was built from the following predictors: IMO, antibiotics before diet, full diet finalization (elimination and reintroduction) and good tolerance of diet, based on the *p* value condition of *p* < 0.25^2^ (antibiotics were included in the model due to close p value level). TSBS levels before the diet was included as a covariate. Multivariate model indicated that antibiotics used before the diet increased odds of higher response 7x, OR = 7.10 CI95 [1.28;44.64], *p* = 0.028. Additionally, full diet finalization (elimination and reintroduction) increased odds of higher response 3x, OR = 3.43 CI95 [1.22;10.51], *p* = 0.024 (Table 5). Multivariate model fit was assessed with Negelkerky R2 with the outcome of 39.6% and Hosmer and Lemeshow test with the outcome of *p* = 0.475, both indicating good model fit. VIF indicators were used to assess collinearity, all variables noted VIF below 2.

## Discussion

Irritable bowel syndrome (IBS) is a chronic functional disorder of the gastrointestinal tract. The prevalence of irritable bowel syndrome (IBS) in Europe and North America, estimated on the basis of population studies, is approximately 10–15%. The pathogenesis of the disease is unknown, but it is likely to be multifactorial and involves: gastrointestinal motility abnormalities, immune disorders of the intestinal mucosa, dysbiosis of gut microbiota, visceral hypersensitivity, central nervous system disorders. The Rome IV criteria define IBS as the presence of abdominal pain occurring at least 1 day a week for the past 3 months, which is associated with at least two of the following symptoms: change in bowel movement frequency, change in stool consistency, and relief or worsening of pain in relation to bowel movements (10). The main symptom of IBS is abdominal pain. This pain is usually described as a feeling of cramping of varying intensity and periodic exacerbations. In addition, patients often report bloating, and studies show a reduced quality of life in these patients, which is associated with the chronic nature of the disease and the inability to fully cure it (11–13).

Based on predominant bowel habits, four types of IBS are distinguished (14): 1. IBS with predominant diarrhea (IBS-D); 2. IBS with predominant constipation (IBS-C); 3. Mixed IBS (IBS-M); 4. Unclassified IBS (IBS-U).

Due to its unknown etiology, targeted treatment of IBS is not possible. The suggested treatment strategy for IBS is a “step-up” approach, i.e., starting with the simplest non-pharmacological modifications and progressing to pharmacological treatment. The importance of establishing good cooperation with the patient and reassuring them that they do not have any serious diseases is emphasized. The next step is to educate the patient on how to avoid situations that exacerbate symptoms, modify their diet, and only if there is no improvement, proceed to pharmacological treatment (15–18).

SIBO is dysbiosis with a small bowel colonized by an excessive number and/or abnormal type of anerobic and anaerobic microbes that are typically found in the colon. Common symptoms of SIBO include flatulence, abdominal discomfort, chronic watery diarrhea, but also constipation. Meta-analyses suggest that 78% of IBS subjects suffer from SIBO (19).

The diagnosis of SIBO is confirmed with a positive carbohydrate breath test or a bacterial concentration of >10^3^ colony forming units/mL in a duodenal aspirate culture. As non-invasive, breath testing is the most common practice (20).

Symptoms similar to SIBO may be caused by an excess of methane-producing microorganisms (methanogenic archaea) and in that situation patients are diagnosed with IMO.

The first-line treatment in the management of SIBO and IMO involves using antibiotic therapy.

In patients with small intestinal bacterial overgrowth, rifaximine is used and in case of IMO, combination of rifaximin and neomycin or metronidazole is recommended.

Alternative antibiotics for treatment of SIBO include trimethoprim-sulfamethoxazole, norfloxacin, tetracycline, ciprofloxacin or amoxicillin-clavulanic acid (19, 20). According to Liébana-Castillo et al. comprehensive approach, combining pharmacotherapy with, appropriate dietary intervention improve quality of life in SIBO patients (21).

The LFD is one of the proposed dietary interventions for patients with IBS. European, American, and Asian scientific societies are very cautious in recommending the LFD (Table 6). They usually recommend it for selected groups of patients, for a short period of time and under the supervision of a dietitian (22–31).

**Table 6:** Dietary recommendations for low FODMAP diet (22–31).

Scientific society	Dietary recommendations
British Society of Gastroenterology (BSG), 2021	A low FODMAP diet is not a first-line treatment. It should be considered if there is no improvement after previously introduced dietary modifications, taking into account lifestyle factors.
United European Gastroenterology and European Society for Neurogastroenterology and Motility (UEG/ESNM), 2022	Short-term use of a low FODMAP diet in patients with diarrhea when other treatments have been ineffective;
Polish Society of Gastroenterology (PTG-E), 2018	Consideration of a low FODMAP diet
American College of Gastroenterology (ACG), 2021	To consider a low FODMAP diet under the supervision of an experienced dietitian
Canadian Association of Gastroenterology Clinical Practice (CAG), 2019	A low FODMAP diet in some patients should be used for a short period of time and under the supervision of a specialized dietitian,
Japanese Society of Gastroenterology (JSGE), 2020	No recommendation for a low FODMAP diet
Danish Society for Gastroenterology and Hepatology (DSGH), 2017	Low FODMAP diet under strict supervision of a dietitian;
Romanian Society of Neurogastroenterology (SRNG), 2021	Short-term use of a low FODMAP diet in patients for whom other treatments have been ineffective. No recommendation for all patients.
Asian Neurogastroenterology and Motility Association (ANMA), 2019	A low FODMAP diet may be helpful in treating

Previous studies have found a reduction in IBS symptoms during a low FODMAP diet compared to placebo (31–33), especially in patients with IBS-D (34). According to Altobelli and Marsh, a LFD could have a favorable impact on IBS symptoms, especially abdominal pain and bloating (7, 35). There remained doubts as to whether the effect of the diet was due to the diet as a whole or to the removal of a single component, e.g., lactose (36). Low FODMAP diet protocol, seems to be a complementary approach to SIBO treatment, through the exclusion of rapidly fermentable, short-chain carbohydrates (37, 38). Redondo-Cuevas et al. compared patients with SIBO or IMO treated with antibiotic therapy and a low-FODMAP diet with a group of patients treated additionally with herbal antibiotics, probiotics, and prebiotics, obtaining similar results in terms of symptoms (39). Jent et al. concluded that low FODMAP diet in IBS improves outcomes compared to a control diet, but due to diverse study designs and heterogeneity of results, a clear superiority of LFD over control diets could not be proven (40). Similarly, Haghbin et al. noted the effectiveness of the low FODMAP diet, but pointed out the need for further RCT studies (41).

At the same time, Zeraattalab-Motlagh et al. did not demonstrate the effectiveness of the LFD in IBS, pointing out the lack of assessment of the long-term effectiveness of the diet (42).

### Study group

IBS is significantly more prevalent in women than men, particularly in the United States and Europe, with ratios of about 2:1 for those seeking medical care (7). In our study, the majority of patients were women. In most studies on the effectiveness of LFD analyzed by Jent et al., also the majority of participants were women, ranging from.

42 to 86%; in seven of the studies analyzed more than two-thirds of the study population were women and no differences were found between women and men in terms of the effectiveness of the diet (40). Xing et al. assessed whether there are gender differences in response to group education on the LFD for irritable bowel syndrome and no differences were found either. Other available research results do not indicate a statistically significant difference in the reduction of IBS symptoms after a LFD diet between women and men (43).

The most common symptoms reported by patients participating in our study corresponded to the most common symptoms described in the available literature (44, 45) and included flatulence, irregular bowel movements and abdominal pain.

### Symptom reduction

According to the ASG (American College of Gastroenterology) Clinical Guideline, the low-FODMAP diet is the most evidence-based dietary intervention for IBS due to its reduction of symptoms. In five meta-analyses on this effect, a statistically significant reduction in symptoms was observed in patients with IBS following a low-FODMAP diet (9, 41, 46–48). At the same time, data from previous studies on the effectiveness of reducing abdominal pain, stool consistency and stool frequency are inconclusive (7, 40–42).

In our study, we found a reduction in symptoms with the low FODMAP diet in almost all of patients. The greatest effect was seen for the most bothersome symptoms, namely bloating, which is consistent with the existing literature (1, 4, 7, 49). An interesting finding is that most of our patients reported constipation rather than diarrhea. This would require further investigation, including analysis of the medications used and foods consumed.

### Diet tolerance

The LFD was well tolerated which is consistent with the existing literature (50). Only 9 people in our study reported poor diet tolerance (including 1 person who reported headaches, dizziness and constipation, while the others reported persistent IBS symptoms despite the diet, which may indicate that they did not understand the question properly).

### Probiotics

In irritable bowel syndrome pathogenesis gut dysbiosis plays significant role, although specific microbial compositional changes and mechanisms are unclear (51). Most of the studies concluded that some diet interventions modify the microbiota in IBS and systematic review demonstrated the efficacy of Lactobacillus in reducing IBS symptoms (46, 52). Previous studies have shown the effectiveness of Lactobacillus or Bifidobacterium in reducing IBD symptoms (3, 52, 53). In our study 64 participants used probiotics during the LFD. These were various preparations with different compositions (including *Bifodobacterium* spp., *Lactobacillus* spp., *Sacharomyces boulardi*) and the use of probiotics during the diet did not have a significant effect on its outcome. Our group of patients was too diverse to draw conclusions. This requires further research on a larger group.

### Antibiotics

Previous publications have suggested that rifaximin reduces IBS symptoms by affecting the gut microbiota, and there are recommendations to rifaximin in IBS with diarrhea symptoms (23, 54, 55). Rifaximin is a non-absorbable antibiotic that inhibits bacterial RNA synthesis and due to its poor absorption has an increased exposure to the intestine locally acting with activity against anaerobic, gram-positive, and gram-negative bacteria. May be used not only in diarrhea but also for the treatment of patients with IBS with mixed bowel habits and in patients with persistent global symptoms (56). In our study, in most cases the low-FODMAP diet was preceded by antibiotic therapy and people who had previously used antibiotics were approximately more likely to respond well to the diet.

### Compliance and dietitian support

Both American and Canadian gastroenterological societies recommend supervision by an experienced dietitian when a patient is following a LFD (Table 7). This is justified by the fact that this diet, especially in phase II, requires knowledge of the symptoms that can be expected when expanding the diet and how to respond to them. Although our patients` results did not differ depending on age, gender or whether the patient had a dietary consultation, more than half of the patients decided to use the support of a dietitian when planning to start a LFD. More than half of the participants rated the diet as difficult to follow and less than half of patients completed the full diet, although completion of both phases was associated with a 3.5-fold increase in the likelihood of symptom resolution. This again highlights the need for support for patients following a low FODMAP diet.

**Table 7:** Outcome of logistic regression analysis for higher response to diet.

Variable	Univariate models (Adjusted for TSBS before diet)			Multivariate model (Adjusted for TSBS before diet)		
	OR	95% CI for OR	*p*	OR	95% CI for OR	*p*
Age, years	1.00	0.94–1.06	0.898	–	–	–
Sex, female (vs male)	1.72	0.17–16.71	0.622	–	–	–
Disease type						
SIBO	0.85	0.25–2.68	0.780	–	–	–
IMO	0.50	0.19–1.26	0.148	0.37	0.12–1.03	0.064
IBS	1.43	0.54–3.78	0.468	–	–	–
Other	0.66	0.10–3.88	0.646	–	–	–
Antibiotics before diet	2.39	0.53–11.45	0.255	7.10	1.28–44.64	**0.028**
Diet split elimination and reintroduction phases*	1.80	0.59–5.66	0.305	–	–	–
Dietary consultation	1.57	0.62–4.02	0.344	–	–	–
Full diet finalized (elimination and reintroduction)	2.55	0.99–6.95	0.058	3.43	1.22–10.51	**0.024**
Good tolerance of diet	4.82	0.64–45.82	0.131	3.64	0.49–34.30	0.210
Probiotics during the diet	0.74	0.28–1.89	0.529	–	–	–

OR, Odds ratio; CI, Confidence interval. All models included value of Total Symptom Burden Score (TSBS) before diet. *Data available for *n* = 96 patients.

### Limitation of the study

The large number of women in our study (95.9%) may be due to the fact that women were more willing to follow the low FODMAP diet recommended by their doctor. There are no data available in the literature on the ratio of women to men in the use of the low FODMAP diet.

The online questionnaire used for this study has not been validated. To our knowledge, there is currently no validated questionnaire available on the effectiveness of the low FODMAP diet, but our questionnaire may serve as pilot material for future validation.

In the questionnaire, patients could tick all symptoms that occurred before the low FODMAP diet, not just the predominant symptom. Therefore, patients with variable bowel movements ticked both diarrhea and constipation. In future surveys, it would be useful to include a separate question about the predominant symptom.

Patients could have also tick a few diagnoses, including IBS and SIBO and IMO. In future studies it would be useful to analyze the influence of multi-morbidity on results of intervention. However, in our study the reduction in symptoms occurred independently of the diagnosis.

In future studies involving a larger number of patients, it would also be necessary to separate out and analyze the group of patients taking antibiotics, considering their effect on the microbiota-gut-brain axis.

## Conclusion

A low FODMAP diet resulted in a significant reduction in reported gastrointestinal symptoms and was well tolerated by patients with IBS or SIBO or IMO. Less than half of the patients reported the diet as easy to follow and only 43.9% of patients completed the low FODMAP diet. The reduction in symptoms occurred independently of the diagnosis. The diet proved difficult to follow, and more than half of the patients sought the support of a dietitian, which confirms the importance of such support. Predictive factors for a good response to a low-FODMAP diet were completion of two phases of the diet and antibiotic treatment prior to starting the diet.

## Data Availability

The raw data supporting the conclusions of this article will be made available by the authors, without undue reservation.

## Footnotes

1 David W. Hosmer, Stanley Lemeshow, Applied Logistic Regression, 2000.

2 David W. Hosmer, Stanley Lemeshow, Applied Logistic Regression, 2000.
